# Advancements in the synthesis of naphthoxazine-3-one derivatives over the last two decades: a brief review

**DOI:** 10.1039/d6ra03522a

**Published:** 2026-05-11

**Authors:** Digambar B. Bankar, Santosh T. Shinde, Kaluram G. Kanade, Dinesh. P. Amalnerkar, Shrikant P. Takle

**Affiliations:** a Post Graduate Department of Chemistry and Research Centre, R. B. Narayanrao Borawake College Shrirampur (Autonomous) 413709 India bankardb100@gmail.com; b Post Graduate Department and Research Centre of Chemistry, Annasaheb Awate College Manchar-410503 India drsantoshinde@gmail.com; c Rajmata Jijau Shikshan Prasarak Mandal's Arts, Commerce & Science College Landewadi, Bhosari Pune-411039 India; d Department of Humanities and Applied Science, Pimpri Chinchwad College of Engineering Nigdi Pune-411044 (Maharashtra) India; e Post Graduate Department and Research Centre of Chemistry, Annasaheb Magar Mahavidyalaya Hadapsar Pune 411028 India

## Abstract

Multicomponent reactions (MCRs) play a significant role in organic synthesis, medicinal chemistry and in material science, as they permit the formation of a single product from the strategic combination of more than two starting materials. Multicomponent reactions (MCRs) represent an attractive area of research for the efficient synthesis of highly functionalized, biological active and structurally diverse heterocyclic organic compounds. Synthesis of naphthoxazine-3-one derivatives is an important multicomponent reaction. Such derivatives are typically prepared in a one-pot from β-naphthol, aromatic aldehydes and urea in the presence of suitable catalyst thereby providing most efficient route for the synthesis of structurally diverse naphthoxazinone derivatives. Aromatic condensed naphthoxazinone derivatives represent an important class of functionalized building blocks. Naphthoxazinone derivatives are well-known by their significant biological activities such as anti-inflammatory, anti-ulcer, antibacterial, antipyretic, antifungal, antihypertensive, *etc.* These compounds also exhibit a wide range of industrial, clinical and pharmacological applications. Several researcher groups have made notable contributions in the field of synthesis of naphthoxazine-3-one derivatives using various catalytic systems. This review highlights the advances made over the past two decades in catalytic approaches, including nanocatalysis, for the synthesis of biologically active naphthoxazine-3-one derivatives. Especially, it emphases on methodologies reported by various researchers involving multicomponent cyclocondensation reactions of β-naphthol, aromatic aldehydes, and urea under specific reaction conditions. Most of the reported protocols have significant advantages like use of non-toxic and inexpensive catalyst, shorter reaction time, solvent-free conditions, easy work-up and purification of compounds, good to excellent yields of the desired naphthoxazine-3-one derivatives, recyclability of the catalysts, making green and environmentally benign protocols. This review will be definitely beneficial for researchers engaged in designing more efficient, novel and environmentally friendly synthetic protocols for the synthesis of naphthoxazine-3-one derivatives as well as for developing new biologically active heterocyclic molecules.

## Abstract 2

Multicomponent reactions (MCRs) represent an attractive area of research for the efficient synthesis of highly functionalized, biological active and structurally diverse heterocyclic organic compounds such as naphthoxazine-3-ones.

## Graphical abstract

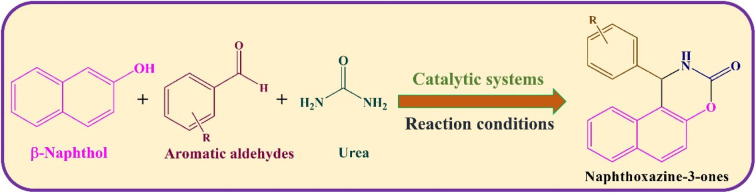

## 1. Introduction

Multicomponent reactions (MCRs) involve the use of three or more reactants to form a single product which incorporates the essential parts of the starting materials. Multicomponent reactions have emerged as a powerful tool in the modern synthetic organic chemistry enabling the facile synthesis of complex molecules in a one-pot reaction without the need for isolation and purification of reaction intermediates. As a result, MCRs reduce cost, time and energy. In addition, multicomponent reactions are modular and convergent in nature, making them a powerful strategy for generating the molecular diversity.^1^

Multicomponent reactions are considered as an innovative strategy in the organic chemistry and hence, they form an important component of sustainable chemistry and represent a new approach to ideal organic synthesis. Moreover, multicomponent reactions are typically safer and more environmentally friendly.^2^

Multicomponent reactions offer advantage like high atom economy and have become essential protocols for generating bioactive molecules and preparing large libraries of small molecules that are valuable for discovering lead compounds in the fields of pharmaceuticals and agrochemicals.^3^

One of the important multicomponent reactions is the synthesis of naphthoxazine-3-one derivatives. Aromatic condensed oxazinones like naphthoxazine-3-ones (naphthoxazinones) are an important class of heterocyclic compounds, because many of these heterocyclic compounds exhibit significant biological activities. Generally, naphthoxazine-3-one derivatives are prepared from β-naphthol, aromatic aldehydes and urea using suitable catalyst and reaction conditions.

Many of aromatic condensed oxazinone heterocyclic compounds exhibit interesting biological properties such as anti-inflammatory, antiulcer, antipyretic, antihypertensive, antifungal^4,5^ and antibacterial properties.^6^ Some of oxazinones and their fused poly nuclear heterocyclic compounds exhibit biological activities such as anti-proliferative,^7^ act as platelet fibrinogen receptor antagonists and calmodulin antagonists,^5^ 5-HT ligand binding inhibitors.^8,9^

Naphthoxazinone derivatives and their analogues display a wide range of important biological properties such as antimalarial, antitumor, antiarrhythmic, HIV inhibitor and anti-viral.^10^

Besides, naphthalene-condensed 1,3-oxazin-3-ones have been used as precursors in the preparation of phosphogenic ligands for the asymmetric catalysis.^11^

Owing to such significant importance of aromatic condensed oxazinone compounds, many researchers have developed diverse synthetic protocols for the synthesis of such compounds using various catalytic systems particularly acidic systems and also nano-catalysis under specific reaction conditions. In recent years, major significant development and advancements have been made in the field of catalysis. In 2022, Zhimomi *et al.* published a concise review highlighting recent developments in green synthetic strategies for the preparation of 1,3-oxazine derivatives. Their work outlines a variety of synthetic approaches employing different starting materials under specific reaction conditions. Notably, the authors summarized nearly 23 methodologies for synthesizing naphthoxazinone derivatives using β-naphthol, aromatic aldehydes, and urea as key precursors. The review emphasizes diversity-oriented synthesis, focusing on green synthetic methods as well as the biological and material relevance of [1,3]-oxazine compounds.^3^

In 2013, Didwagh and Piste reported a short review on the green synthesis of thiazine and oxazine derivatives, focusing on environmentally friendly strategies for their preparation. Their review highlights advancements in sustainable approaches, including solvent-free methods, microwave irradiation, sonication, grinding technique, nanoparticles, ionic liquids, *etc*, based on literature published up to 2012.^12^

Naphthoxazine-3-one derivatives exhibit wide range of biological applications. Consequently, numerous procedures for their synthesis have been developed from time to time, with increasing emphasis on green and environmentally benign approaches. In this review article, our aim is to systematically describe the synthetic protocols developed by various researchers for the synthesis of naphthoxazine-3-one derivatives from β-naphthol, aromatic aldehydes and urea over the last two decades. This review enables researchers to readily recognize the significant advancements made in the synthesis of biologically important naphthoxazine-3-one derivatives and is expected to facilitate the development of new and innovative synthetic protocols for the synthesis of naphthoxazine-3-one derivatives.

## 2. Synthesis of naphthoxazine-3-one derivatives using various catalytic systems

### 2.1. P-TSA mediated synthesis of naphthoxazine-3-one derivatives

**Scheme 1:**
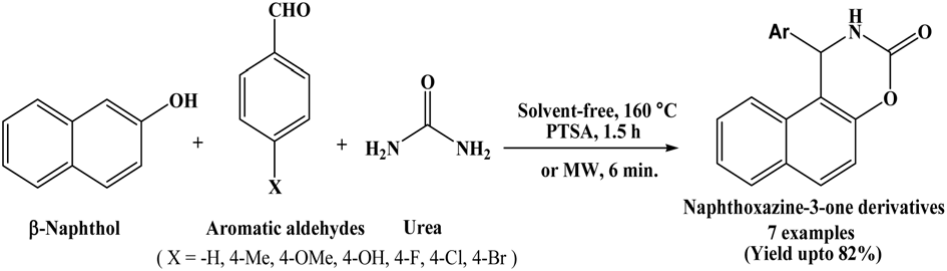
Synthesis of naphthoxazine-3-one derivatives catalyzed by P-TSA.

In 2007, Dabiri *et al.* have developed novel and facile protocol for the synthesis of 1,2-dihydro-1-arylnaphtho[1,2-e][1,3]oxazine-3-one derivatives from the cyclocondensation of β-naphthol, aromatic aldehydes and urea employing p-TSA as a catalyst under solvent-free conditions at 160 °C, which afforded good yields of the products (Scheme 1)^13^ in 1.5 h. To identify the most effective catalyst, the authors carried out the synthesis of naphthoxazinone using β-naphthol, benzaldehyde, and urea under similar reaction conditions. The reactions were performed both in the absence of a catalyst and in the presence of various catalysts such as AcOH, P-TSA, LiCl, CuCl_2_, and NiCl_2_. Among these, P-TSA afforded the highest yield of the desired product. In order to decrease the reaction time, the condensation of β-naphthol, aromatic aldehydes and urea was accomplished under solvent-free and microwave irradiation conditions without using any acidic catalyst, that could be another advantage of this protocol in the synthesis of naphthoxazinones. Under microwave irradiation, reaction time decreased from 1.5 h to 6 minutes with increased yields of corresponding naphthoxazine-3-one derivatives compared to conventional heating method.

### 2.2. Catalyzed by [bmim]Br/p-TSA

**Scheme 2:**
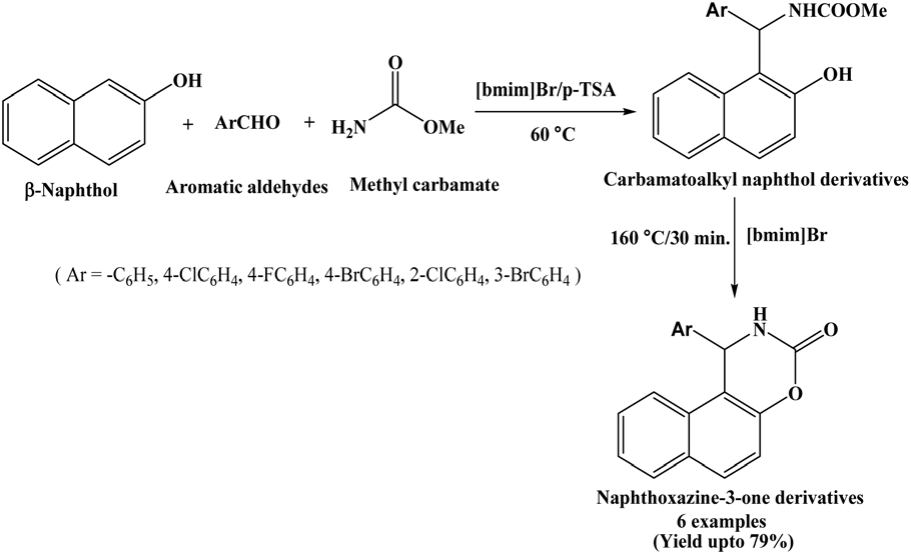
Synthesis of naphthoxazine-3-one derivatives using [bmim]Br/p-TSA.

Ionic liquids are low melting point salts with inorganic anion and organic cation. They have attracted significant attention due to their unique properties, including negligible vapor pressure, non-flammability, excellent electrochemical stability, efficient ion transport, and good ion mobility and miscibility with a wide range of organic materials. These distinctive characteristics make ionic liquids highly promising for diverse applications across various fields.^14^ Dabiri *et al.* in 2007 synthesized carbamatoalkyl naphthol derivatives in good yields by the condensation of β-naphthol, aromatic aldehydes and methyl carbamate in a one-pot using p-TSA in [bmim]Br as an ionic liquid at 60 °C. The carbamatoalkyl naphthol derivatives were efficiently converted into naphthoxazinones by heating at 160 °C in [bmim]Br solvent within 30 minutes in absence of any catalyst (Scheme 2).^15^ The corresponding naphthoxazinones were obtained in good yields (up to 79%).

### 2.3. Catalyzed by zinc triflate, Zn(OTf)_2_

**Scheme 3:**
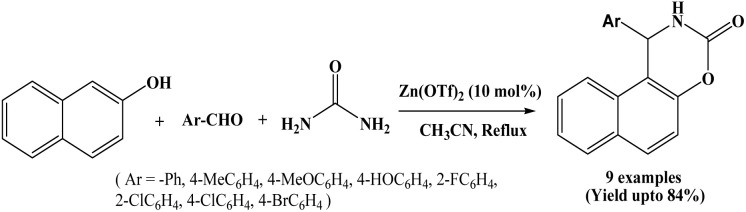
Zinc triflate mediated synthesis of naphthoxazine-3-one derivatives.

In 2009, Hajra and co-workers reported the simple and efficient protocol for the one-pot synthesis of naphthoxazinone derivatives from the reaction of β-naphthol, aromatic aldehydes and urea using zinc triflate, Zn(OTf)_2_ (10 mol%) as a Lewis acid catalyst in refluxing acetonitrile solvent (Scheme 3).^16^ For optimization of catalyst, authors carried out the coupling reaction of β-naphthol, benzaldehyde and urea using different catalysts like Zn(OTf)_2_, La(OTf)_3_, In(OTf)_3,_ ZnCl_2_, and Zn(ClO_4_)_2_. Among the tested catalysts, Zn(OTf)_2_ afforded the highest yield. A wide range of aromatic aldehydes with variety of substituents were employed, affording the corresponding naphthoxazinone derivatives in good yields in 5 to 7 h. Piperonal also afforded 74% yield. In the stated protocol, 4-aryl-3,4-dihydronaphtho[2,1-e][1,3]oxazin-2-ones were also synthesized in good yield using α-naphthol in place of β-naphthol.

### 2.4. Synthesis of naphthoxazine-3-ones using TMSCl/NaI

**Scheme 4:**
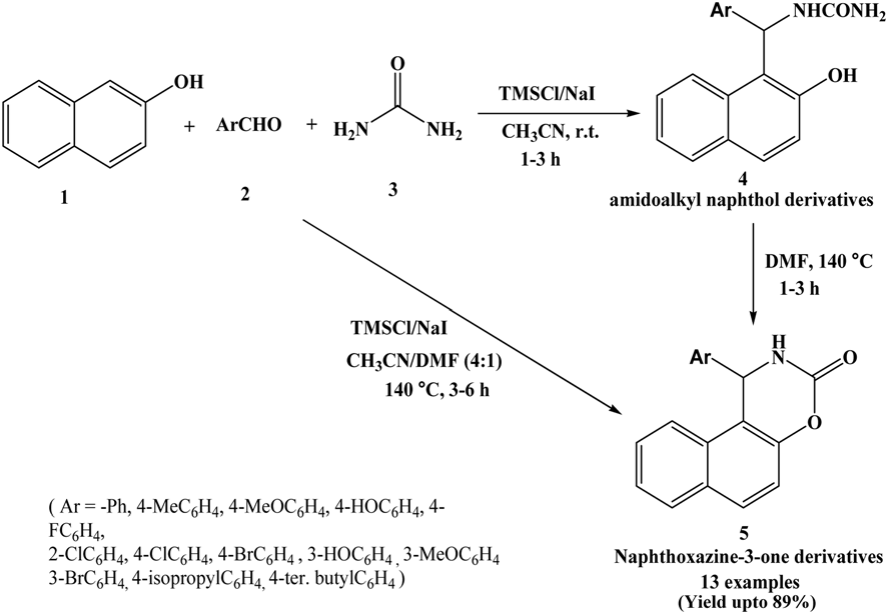
TMSCl/NaI catalyzed synthesis of naphthoxazine-3-ones.

In 2010, Sabitha *et al.* have developed a novel method for the synthesis of naphthoxazine-3-one derivatives from β-naphthol, aryl aldehydes and urea using TMSCl/NaI catalyst at 140 °C in CH_3_CN/DMF (4 : 1) solvent system (Scheme 4).^17^ In a one-pot condensation of β-naphthol, aryl aldehydes and urea, amidoalkyl naphthol derivatives (4 in Scheme 4) have been prepared in good yields using TMSCl/NaI at room temperature in CH_3_CN solvent. At 140 °C temperature, ring closure of amidoalkyl naphthol derivatives occurs, resulting in the formation of naphthoxazine-3-one derivatives (5 in Scheme 4). Authors employed various aromatic aldehydes with substituents such as –Cl, –F, –Br, –OH, –OMe, –CH_3_, isopropyl, *t*-butyl at ortho, meta and para positions.

### 2.5. Synthesis of naphthoxazine-3-ones catalyzed by iodine

**Scheme 5:**
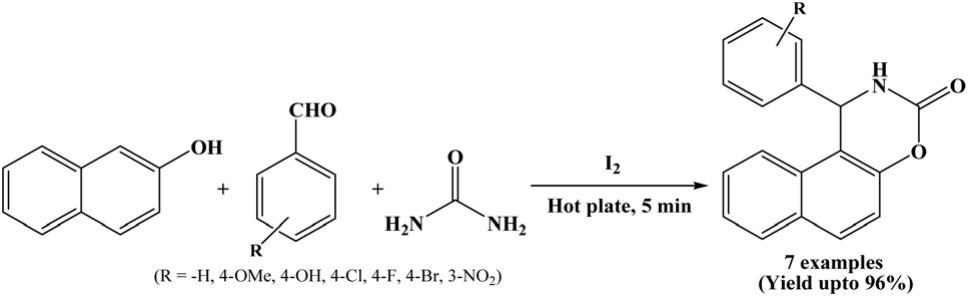
I_2_ mediated synthesis of naphthoxazinones.

Nizam and Päsha in 2010, have developed a methodology using iodine as a catalyst for the synthesis of 1,2-dihydro-1-aryl-naphtho[1,2-e][1,3]oxazine-3-ones from β-naphthol, aryl aldehydes and urea under solvent-free conditions (Scheme 5).^18^ The reactions were carried out at 80 °C on preheated hot plate and were completed within 5 minutes, affording excellent yields of the corresponding naphthoxazinones (90 to 96%). Simplicity and efficiency, shorter reaction time, solvent-free condition are the advantages of the stated protocol.

### 2.6. Silica-gel catalyzed approach for the synthesis of naphthoxazinones

**Scheme 6:**
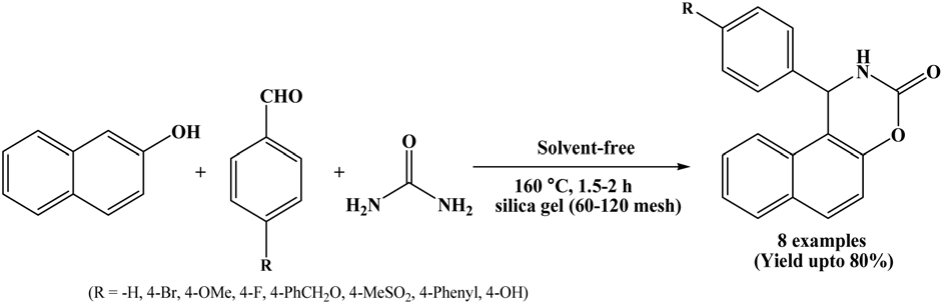
Synthesis of naphthoxazine-3-ones using silica gel (60–120 mesh).

In 2010, Kottawar and co-workers have investigated a method for the synthesis of naphthoxazinone derivatives by the one-pot three component condensation of β-naphthol, aromatic aldehydes and urea using silica gel (60–120 mesh) at 160 °C under solvent-free conditions (Scheme 6).^19^ The reactions were carried out using 30% by wt. of the silica-gel, and reactions completed within 1.5 to 2 h. In this protocol, various naphthoxazinone derivatives were synthesized from wide range of 4-substituted benzaldehydes.

### 2.7. HClO_4_/SiO_2_ catalyzed protocol for the synthesis of naphthoxazine-3-ones

**Scheme 7:**
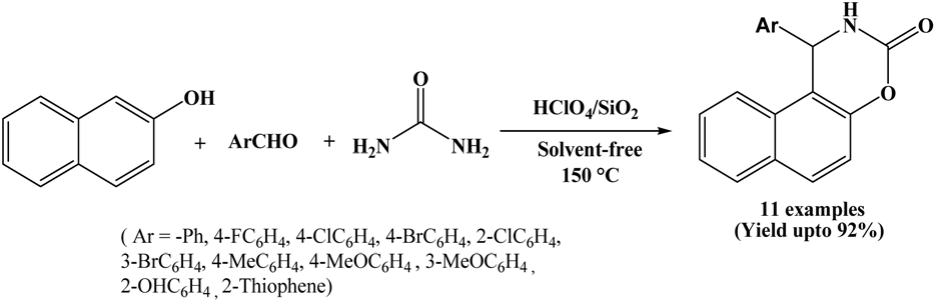
HClO_4_/SiO_2_ catalyzed synthesis of naphthoxazine-3-one compounds.

Facile synthesis of naphthoxazine-3-one derivatives by perchloric acid supported on silica (HClO_4_/SiO_2_) catalyzed one-pot condensation of β-naphthol, aromatic aldehydes and urea has been suggested by Abbastabar Ahangar *et al.* in 2010 (Scheme 7).^20^

A variety of electronically different aromatic aldehydes were studied in their protocol and the corresponding naphthoxazine-3-one derivatives obtained in higher yields under shorter reaction times at 150 °C. Reactions underwent efficiently using 2 mol% of the HClO_4_/SiO_2_ catalyst. Here, easy work-up, low cost, ready availability of the catalyst, solvent-free condition, reusability of the catalyst and simple isolation of the product are the additional advantages.

### 2.8. Catalyzed by phosphomolybdic acid, (H_3_Mo_12_O_40_P)

**Scheme 8:**
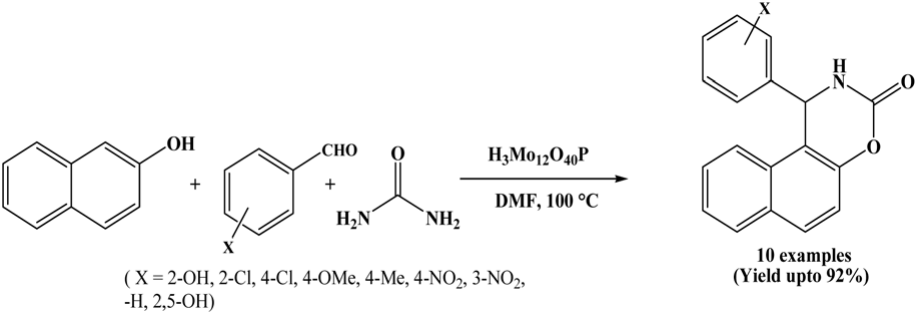
Synthesis of naphthoxazine-3-ones over phosphomolybdic acid.

In 2011, Chaskar *et al.* reported novel and efficient synthesis of 1,2-dihydro-1-aryl-3H-naphth[1,2-e][1,3]oxazin-3-one derivatives using phosphomolybdic acid (H_3_Mo_12_O_40_P) as a recyclable catalyst. The β-naphthol, aromatic aldehydes and urea underwent the reaction in DMF solvent at 100 °C to afford the corresponding products in good to excellent yields (Scheme 8).^21^ For comparative study, authors have employed various organic solvents such as EtOH, MeOH, DMF, CH_3_CN, IPA, CHCl_3_, toluene and CH_2_Cl_2_. However, DMF has been proven the best solvent in terms of yield. The authors extended the scope of the reaction by employing various aryl aldehydes containing both electron-donating and electron-withdrawing substituents. In all these examples, observed better yield results regardless the nature of substituents. Furthermore, disubstituted aromatic aldehydes were examined. One substrate contained hydroxyl groups at the 2- and 5-positions, while another possessed a hydroxyl group at the 2-position and a methoxy group at the 5-position. For both of these substrates, the reaction proceeded efficiently, affording around 85% yields of corresponding products. In the present protocol, the catalyst could be recovered and reused as a heterogeneous catalyst.

### 2.9. TMSCl (chlorotrimethylsilane) catalyzed approach

**Scheme 9:**
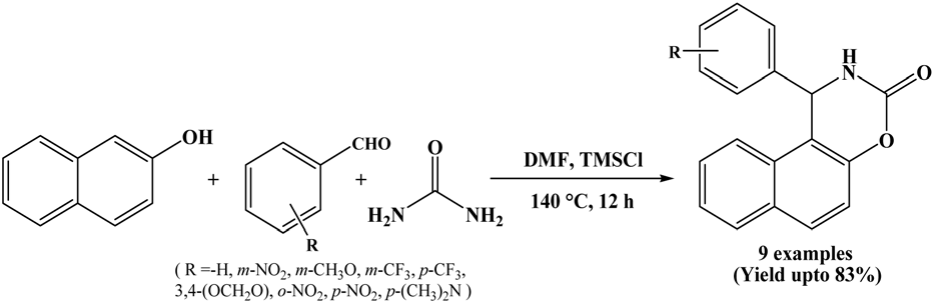
TMSCl catalyzed synthesis of naphthoxazine-3-ones.

In 2010, Jiang *et al.* have reported that the 1,2-dihydro-1-arylnaphtho[1,2-e][1,3]oxazine-3-one derivatives were obtained by the TMSCl catalysed multicomponent condensation of 2-naphthol, aromatic aldehydes and urea. Reactions performed in DMF solvent at temperature of 135–140 °C and afforded the corresponding compounds in 49–83% yields in 12 h (Scheme 9).^22^ The use of inexpensive and relatively non-toxic TMSCl reagent as a catalyst is the silent feature of this protocol.

### 2.10. Montmorillonite K10 clay catalyzed protocol

**Scheme 10:**
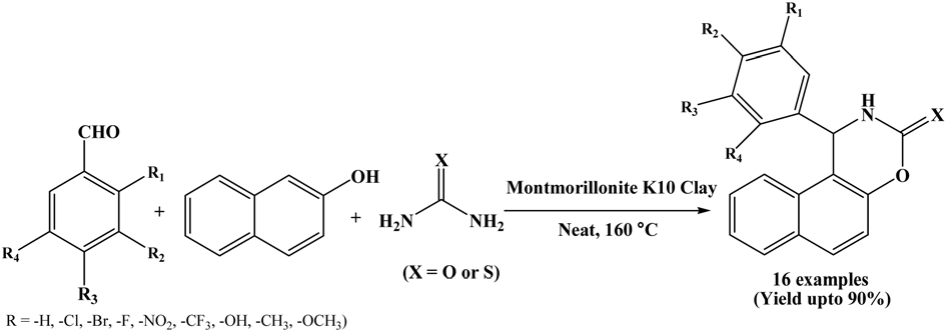
Synthesis of naphthoxazinones using montmorillonite K10 clay.

An efficient green protocol has been explored by Kantevari *et al.* in 2010 for synthesizing naphthoxazinone derivatives from Montmorillonite K10 clay as a heterogeneous catalyst (Scheme 10).^23^ One-pot three component reactions of β-naphthol, aromatic aldehydes and urea/thiourea were performed under solvent-free conditions at 160 °C in shorter reaction times (30 to 90 minutes). A wide range naphthoxazinones were synthesized simply by varying aromatic aldehydes. The authors also used heterocyclic aldehydes like furfural and 2-chloroquinoline-3-aldehyde but unfortunately corresponding products were isolated only in 10 to 15% yields. The use of inexpensive and readily available catalyst, mild heterogeneous reaction conditions, shorter reaction time and easy work up are the superior features of this protocol.

### 2.11. Synthesis of naphthoxazinones *via* copper NPs catalysis

**Scheme 11:**
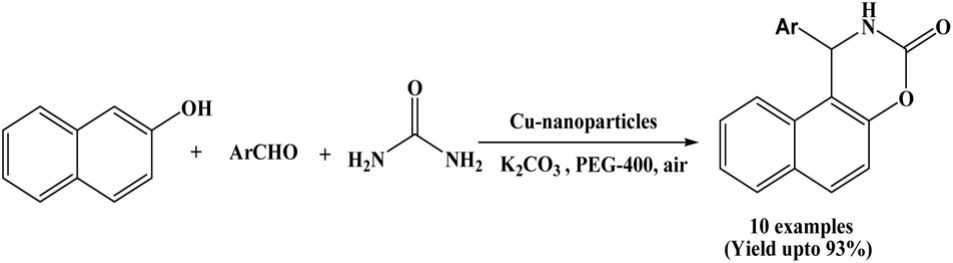
Cu nanoparticles mediated synthesis of naphthoxazinones.

In 2011, Kumar *et al.* have reported an efficient methodology for the synthesis of 2-naphthol condensed 1,3-oxazinone derivatives using copper nanoparticles in the presence of K_2_CO_3_ as a base in PEG-400 solvent at room temperature under air atmosphere (Scheme 11).^5^ In the reported protocol, a range of aryl aldehydes bearing electron-donating and electron-withdrawing substituents, as well as furfural, were employed in the synthesis of naphthoxazinones, affording the desired products in better yields. This protocol offers many advantages such as high yields, clean reaction, short reaction time, recyclability of the catalyst and simple workup procedure making the protocol greener and environmentally benign. The copper nanoparticles and PEG-400 could be recycled for five cycles with negligible loss of their activity. It is suggested that the PEG not only acts as a green and environmentally benign medium but also provides stability to the nanoparticles, preventing further oxidation.

### 2.12. Catalyzed by thiamine hydrochloride

**Scheme 12:**
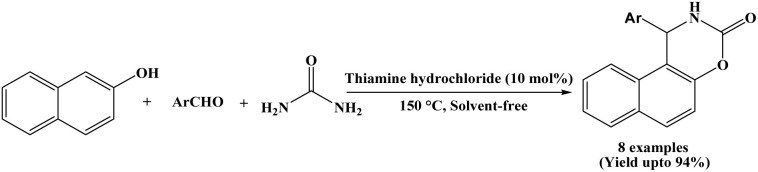
Synthesis of naphthoxazinones catalyzed by thiamine hydrochloride.

In 2011, Lei *et al.* reported a chemoselective three-component condensation reaction of β-naphthol, aromatic aldehydes and urea for the synthesis of naphthoxazine-3-ones under solvent-free conditions, using 10 mol% of thiamine hydrochloride (VB1) as a catalyst (Scheme 12).^24^ This protocol affords the desired products in good to excellent yields (83–94%) within 30 minutes at 150 °C. In the stated protocol, the authors selected a series of aldehydes to undergo condensation reaction with β-naphthol and urea under solvent-free condition. Aromatic aldehydes bearing substituents like –H, –CH_3_, –OCH_3_, –Cl reacted smoothly and afforded the corresponding products in excellent yields (85–94%). Aromatic aldehydes bearing electron withdrawing groups such as –NO_2_, –CN and Cl produced corresponding naphthoxazinones in good yields (83–92%) within 30 minutes at 150 °C.

### 2.13. Catalyzed by ZnO nanoparticles

ZnO nanoparticles have attracted considerable attention because of their desirable physicochemical properties and wide-ranging applications in various fields including gas sensors,^25^ photocatalysis, biological probes, antibacterial agents and solar cells,^26^ heterogeneous catalyst,^27^ cosmetics, paints, fibers, and it can also act as a Lewis acid in various organic transformations.^28^

**Scheme 13:**
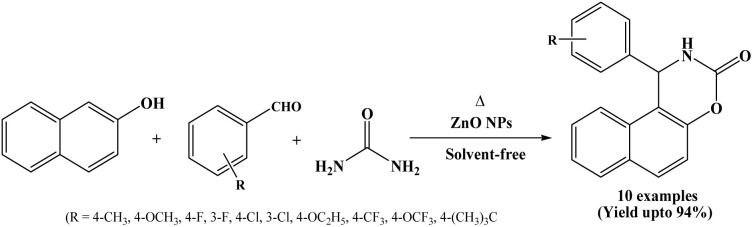
Synthesis of naphthoxazine-3-ones over ZnO NPs.

Rao *et al.* in 2012 have developed an efficient and environmentally benign synthetic route for the synthesis of 1,2-dihydro-1-arylnaphtho[1,2-e][1,3]oxazine-3-ones from aromatic aldehydes, β-naphthol and urea using ZnO nanoparticles under solvent-free and thermal conditions (Scheme 13).^28^ The reactions were carried out at 150 °C and the corresponding naphthoxazinone derivatives were obtained in high yields within a short reaction time (45 to 120 minutes). In the stated protocol, ZnO nanoparticles were used as a heterogenous catalyst and could be reused for four successive cycles. The authors also reported the synthesis of 14-substituted-14H-dibenzo[*a*,*j*]xanthene derivatives catalyzed by ZnO nanoparticles under thermal and solvent-free conditions.

### 2.14. Catalyzed by FeCl_3_/SiO_2_ nanoparticles

**Scheme 14:**
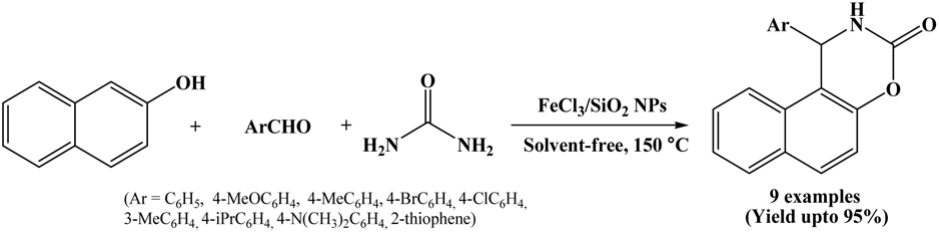
Synthesis of naphthoxazine-3-ones over FeCl_3_/SiO_2_ NPs.

A three-component one-pot condensation reaction of 2-naphthol, aromatic aldehydes and urea in the presence of nano silica supported ferric chloride (FeCl_3_/SiO_2_ NPs) as a green and efficient catalyst was reported by Safaei-Ghomi *et al.* in 2012 (Scheme 14).^29^ To evaluate the catalytic efficiency of FeCl_3_/nano-SiO_2_, the authors conducted a model reaction using benzaldehyde, 2-naphthol, and urea at 150 °C under solvent-free conditions. The activity of FeCl_3_/nano-SiO_2_ was compared with FeCl_3_, nano-SiO_2_, and FeCl_3_/SiO_2_. The results showed that nano silica supported ferric chloride provided the best performance in terms of shorter reaction time and higher yield of corresponding product. This enhanced catalytic activity was ascribed to the nano-SiO_2_ support, enhancing contact surface of materials.

The electron-donating and withdrawing groups containing aromatic aldehydes reacted well under solvent-free conditions at 150 °C and offering corresponding naphthoxazine-3-one derivatives in good to excellent yields within 7 to 20 minutes. This protocol has advantages like shorter reaction time, simple work-up, excellent yield, environmentally benign and catalyst could be reused for five times without any significant loss of catalytic activity making the process greener and more economical.

### 2.15. Wet cyanuric chloride assisted synthesis of naphthoxazine-3-one derivatives

**Scheme 15:**
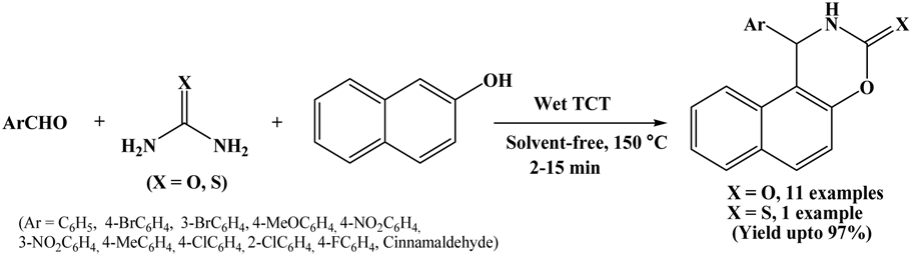
Wet TCT mediated synthesis of naphthoxazinones.

In 2013, Nemati and Beyzai reported a facile protocol employing 10 mol% wet cyanuric chloride (wet TCT) as a catalyst for the synthesis of naphthoxazine-3-one derivatives by one-pot three component condensation of 2-naphthol, aromatic aldehydes and urea under solvent-free conditions at 150 °C (Scheme 15).^30^ The scope of the reaction was extended by using variety of aromatic aldehydes with electron-donating and electron-withdrawing groups and observed good to excellent yields (71–97%) of corresponding naphthoxazine-3-one derivatives. Authors also used thiourea instead of urea and observed 75% yield of the corresponding product. Several advantages of this protocol include high yields, simple procedure, solvent-free conditions easy work-up and short reaction time (2 to 15 minutes).

### 2.16. Catalyzed by silica supported pressler heteropoly acid, H_14_[NaP_5_ W_30_O_110_]/SiO_2_

**Scheme 16:**
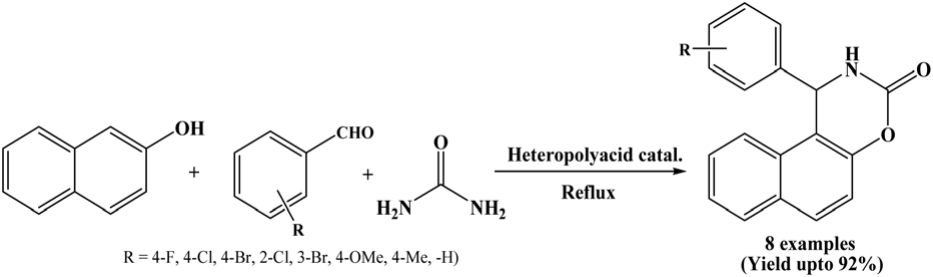
Pressler heteropoly acid catalyzed preparation of naphthoxazinones.

Efficient condensation of β-naphthol, aromatic aldehydes and urea was carried out by Gharib *et al.* in 2013 using silica supported Pressler heteropoly acid as a heterogeneous catalyst under reflux conditions in order to afford the corresponding naphthoxazinone derivatives in good yields in 30 to 130 minutes. The catalyst could be reused for four cycles without appreciable loss in its catalytic activity (Scheme 16).^31^ Operational simplicity, relatively short reaction times, environment friendly aspect, easy handling and heterogeneous nature of the catalyst as well as easy work-up procedure are the notable advantages of this protocol.

### 2.17. Guanidine hydrochloride catalyzed synthesis of naphthoxazinones

**Scheme 17:**
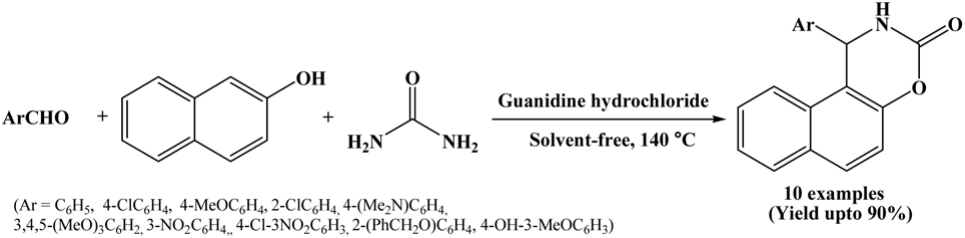
Guanidine hydrochloride mediated synthesis of naphthoxazinones.

In 2013, Olyaei *et al.* have reported a novel method for the synthesis of naphthoxazinones from β-naphthol, aromatic aldehydes and urea catalyzed by guanidine hydrochloride under solvent-free conditions at 140 °C temperature (Scheme 17).^32^ Organocatalysts have been widely employed in various reactions as mono- and bifunctional catalysts due to their economic and environmental advantages. Among the many organocatalysts, hydrogen-bonding compounds like guanidine derivatives are emerging as a powerful tool for the activation of the carbonyl functionality in organic transformations. The naphthoxazinone derivatives were prepared successfully using guanidine hydrochloride as an organocatalyst under solvent-free conditions in short reactions times (60 to 75 minutes) with high to excellent yields making the process more economic and environmentally benign. In the reported protocol, wide range of aryl aldehydes carrying electron-donating and electron-withdrawing substituents were employed in the synthesis of naphthoxazinones, affording the desired products in high to excellent yields.

### 2.18. Catalyzed by pyridinium-based ionic liquid [PyPS][HSO_4_]

**Scheme 18:**
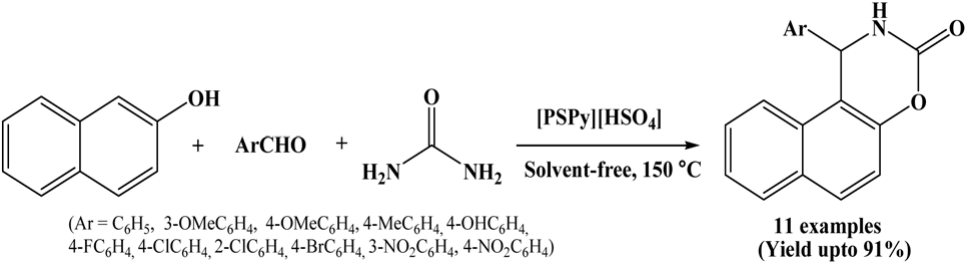
[PyPS][HSO_4_] catalyzed synthesis of naphthoxazinones.

Synthesis of 1,2-dihydro-1-arylnaphtho[1,2-e][1,3]oxazine-3-one derivatives *via* the one-pot multicomponent condensation of β-naphthol, aromatic aldehydes and urea under solvent-free conditions using pyridinium-based functionalized ionic liquids as a novel catalyst has been reported by Dong *et al.* in 2013 (Scheme 18).^33^

The authors screened some pyridinium-based ionic liquids with different anions such as [PSPy][HSO_4_], [PyPS][H_2_PO_4_], [PyPS]p-TSA, [PyPS][BF_4_] and [PyPS][PF_6_] for their ability to catalyze the model reaction of 2-naphthol, benzaldehyde and urea under different thermal conditions.

Out of various pyridinium-based ionic liquids, [PSPy][HSO_4_] offers good results at 150 °C and is a halogen-free ionic liquid which might be more eco-friendly and less expensive. Hence, [PSPy][HSO_4_] has been selected as a catalyst and performed further reactions using the same at 150 °C under solvent-free conditions for the synthesis of napthoxazinone derivatives. This protocol offered good to excellent yields of the corresponding products.

Various solvents such as DMF, ethanol, acetonitrile, dichloromethane and water were also screened for the model reaction, however, good results were achieved only under solvent-free conditions. The present protocol is environmentally benign and offers advantages such as short reaction time, good yields and operational simplicity.

### 2.19. Synthesis of naphthoxazinone derivatives using TiCl_4_

**Scheme 19:**
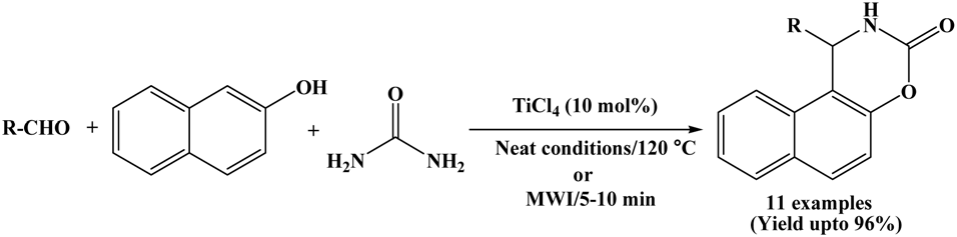
TiCl_4_ catalyzed synthesis of naphthoxazinone derivatives.

Hunnur *et al.* in 2017 reported an economical and efficient catalytic method for the synthesis of naphthoxazinone derivatives using TiCl_4_ (10 mol%) through a one-pot condensation of β-naphthol, aromatic aldehydes and urea by conventional as well as microwave irradiation method (Scheme 19).^34^ The significant reduction in reaction time from 50 to 70 minutes to within 5 to 10 minutes proved the advantage of microwave-assisted method over conventional heating method. Benzaldehyde and a range of aromatic aldehydes bearing both electron-withdrawing and electron-donating substituents were successfully employed, affording the corresponding naphthoxazinone derivatives in good to excellent yields (up to 96%). The reported method is novel, efficient and convenient for the synthesis of corresponding products in good to excellent yields.

In addition to the synthetic study, the authors also performed drug score analysis of the synthesized compounds to evaluate their pharmacological properties. The results indicated that the compounds are generally safe drug candidates, without any tumorigenic, irritability and reproductive effects as evaluated in Osiris property explorer. However, a slight mutagenic property was observed for these compounds.

### 2.20. Catalyzed by silica bonded vanadic acid [SiO_2_–VO(OH)_2_], SVA catalyst

**Scheme 20:**
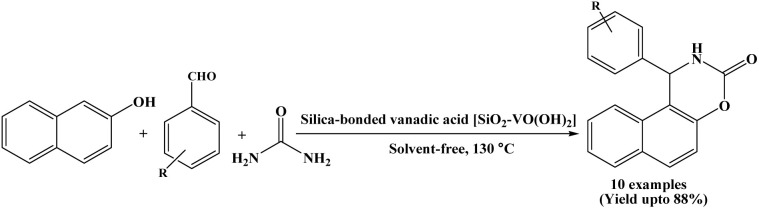
Synthesis of naphthoxazinones over silica bonded vanadic acid catalyst.

In 2015, Zolfigol *et al.* have reported the synthesis of naphthoxazinone derivatives using SVA as a heterogeneous catalyst *via* condensation of β-naphthol, aromatic aldehydes and urea under solvent-free conditions at 130 °C (Scheme 20).^35^ In the present protocol, catalyst was recycled and reused for seven times without any significant loss of catalytic activity. Besides, the authors also synthesized 2,4,6-triaryl pyridine derivatives using SVA through the condensation of aromatics aldehydes, various acetophenones and ammonium acetate. The significant features of the present procedure are short reaction time, high yields, easy work-up and recyclability of the SVA catalyst.

### 2.21. Catalyzed by Fe_2_O_3_ nanoparticles

**Scheme 21:**
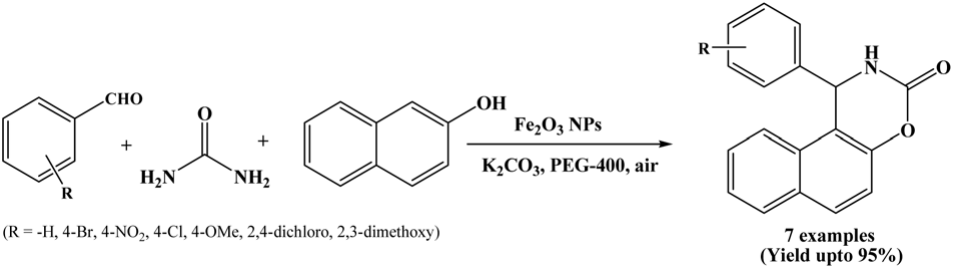
Synthesis of naphthoxazinones over Fe_2_O_3_ nanoparticles.

One-pot synthesis of napthoxazinone derivatives using Fe_2_O_3_ nanoparticles as a heterogeneous acid catalyst was reported by Hashemzehi-Goonaki and Saffari in 2015. The reactions were performed using PEG-400 solvent, K_2_CO_3_ as a base at room temperature under air atmospheric conditions (Scheme 21).^4^

In the present protocol, Fe_2_O_3_ nanoparticles were prepared by microwave assisted method, which confirm a pure Rhombohedral cubic phase, exhibit ferromagnetic behaviour and can be used as a heterogeneous catalyst in the synthesis of variety of napthoxazinone derivatives in high yields. The stated methodology is an efficient and environment friendly for the synthesis of napthoxazinone derivatives.

### 2.22. Catalyzed by silica-bonded S-sulfonic acid

**Scheme 22:**
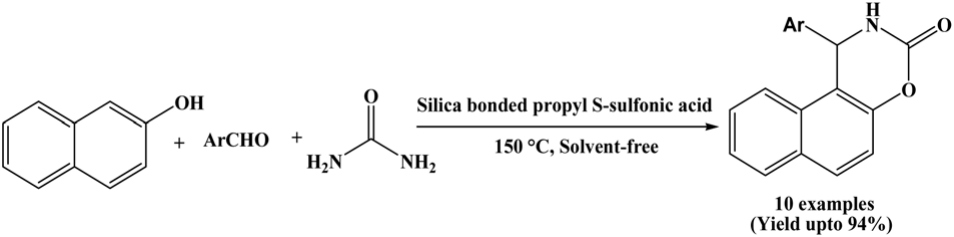
Silica-bonded S-sulfonic acid catalyzed synthesis of naphthoxazine-3-ones.

In 2015, Niknam and Abolpour developed a methodology for synthesizing naphthoxazinone derivatives *via* the cyclocondensation reaction of β-naphthol, aromatic aldehydes and urea using silica-bonded S-sulfonic acid at 150 °C under solvent-free conditions (Scheme 22).^36^ A wide range of aromatic aldehydes bearing electron-donating substituents (R = 3-Me, 4-Me, 4-iso-pr, 4-OMe) and electron-withdrawing substituents (R = 4-Cl, 4-Br, 4-F, 3-F, 3-NO_2_) were employed, affording the corresponding naphthoxazinone derivatives in good to excellent yields (80 to 94%). Silica-bonded propyl-S-sulfonic acid efficiently catalyzed the three-component reactions within 45 to 90 min, and the catalyst was recycled for five consecutive runs without any noticeable loss of catalytic activity.

### 2.23. Catalyzed by CuFe_2_O_4_ nanoparticles

**Scheme 23:**
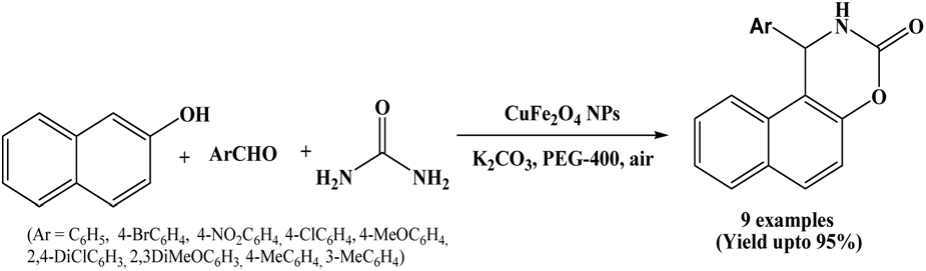
Synthesis of naphthoxazine-3-ones on CuFe_2_O_4_ NPs.

Nanosized ferrite particles are of great interest because of their unique optical, electrical, and magnetic properties. In 2016, Ghaani and Saffari synthesized CuFe_2_O_4_ nanoparticles using microwave-assisted co-precipitation method. The obtained CuFe_2_O_4_ nanoparticles were employed as a nano-heterogeneous, novel, and efficient catalyst for the one-pot three component synthesis of arylnaphtho [1,2-e] [1,3] oxazine-3-one derivatives from β-naphthol, aromatic aldehydes and urea in the presence of K_2_CO_3_ and PEG-400 under ambient reaction conditions (Scheme 23).^37^ Easy work-up, no need to column chromatography, readily available precursors, short reaction times, high yields of the desired products and reusability of the catalyst are the notable advantages of this protocol.

### 2.24. Perlite-SO_3_H NPs mediated synthesis

**Scheme 24:**
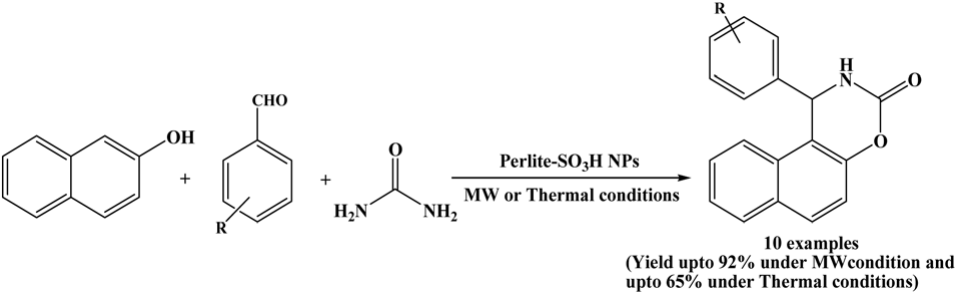
Synthesis of naphthoxazinones using perlite-SO_3_H nanoparticles.

An interesting one-pot three-component strategy for the synthesis of 1,2-dihydro-1-aryl-naphtho[1,2-e][1,3]oxazine-3-one derivatives was developed by Ramazani *et al.* in 2016. In this work, β-naphthol, aromatic aldehydes and urea were reacted with perlite-SO_3_H nanoparticles to furnish naphthoxazinone derivatives in a one-pot under both microwave-assisted and thermal solvent-free conditions (Scheme 24).^38^ Under microwave irradiation (in a microwave oven, operating power of 900 W), the naphthoxazinone derivatives were obtained in greater yields and in shorter reaction times (only 2–10 minutes) compared to the thermal conditions. The catalyst showed good reusability and stability, making the protocol environmentally friendly. It was found that perlite-SO_3_H nanoparticles could be reused for four consecutive cycles with negligible loss of catalytic activity. Additionally, single-crystal X-ray structure analysis and theoretical studies for naphthoxazine-3-one derivative synthesized from β-naphthol, para-isopropyl benzaldehyde and urea were investigated.

### 2.25. Catalyzed by nano-TiO_2_

TiO_2_ possesses several unique characteristics including optical and electrical properties, high photocatalytic activity, non-toxicity, thermal and chemical stability.^39,40^ Owing to these features, TiO_2_ is widely used in many fields such as optoelectronics, photovoltaics, fuel cells, photocatalysis and battery technology. The study on triggerable ion release in polymerized ionic liquids demonstrates that incorporation of thermally labile Diels–Alder linkages enable controlled, on-demand release of mobile ions upon heating, thereby significantly enhancing ionic conductivity. Such tunable ion transport properties are highly relevant for advanced energy systems, as they can improve electrolyte performance in fuel cells, batteries, and photovoltaic devices by enabling responsive control over charge mobility and overall device efficiency.^41^

**Scheme 25:**
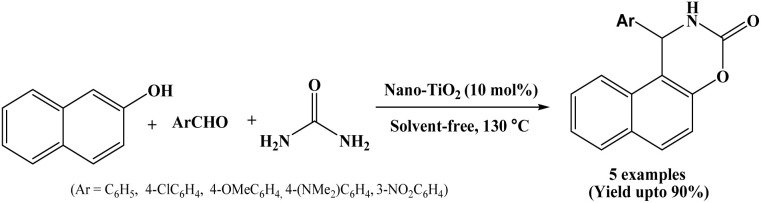
Synthesis of naphthoxazinones over nano-TiO_2_.

In recent years, nano-TiO_2_ has attracted considerable attention as catalyst in the organic synthesis due to its environmental compatibility, ease of handling, chemical stability under high temperature, low cost and non-toxic nature.^42^ In 2018, Pourshamsian reported the synthesis of naphthoxazine-3-one derivatives under solvent-free conditions using nano TiO_2_ as an efficient, heterogeneous clean and eco-friendly catalyst. β-Naphthol, aryl aldehydes and urea undergo cyclocondensation reaction at 130 °C in presence of nano-TiO_2_ catalyst, offering corresponding naphthoxazine-3-ones in good yields under solvent-free conditions (Scheme 25).^42^ The solid nano-TiO_2_ catalyst could be reused for six times without any significant loss of catalytic activity. The method provided several advantages such as short reaction times, good product yields, simple work-up, and environmentally benign conditions. In addition to the synthetic work, the synthesized naphthoxazine-3-one derivatives were tested for antibacterial activity, and the results showed that compounds exhibited significant antibacterial activity, indicating their potential as highly potent antibacterial agents.

### 2.26. Graphine oxide mediated synthesis of naphthoxazinone derivatives

**Scheme 26:**
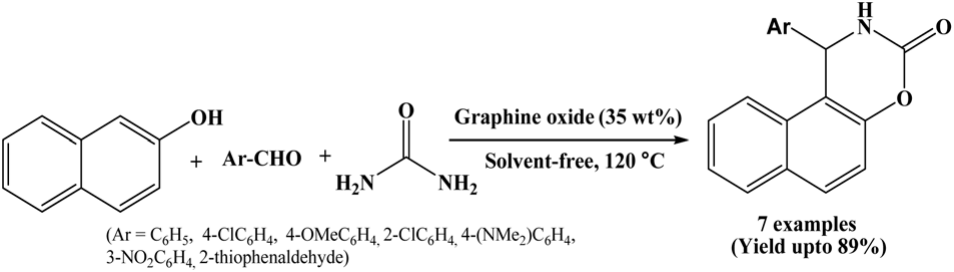
Graphene oxide catalysed synthesis of naphthoxazinones.

Graphene oxide (GO) has emerged as a promising, economical, and efficient carbocatalyst in organic synthesis, due to its unique structural features and versatile surface functionality. In 2016, Gupta *et al.* have investigated the catalytic activity of graphene oxide for one-pot multicomponent synthesis of naphthoxazinone derivatives under solvent-free conditions at 120 °C (Scheme 26).^43^ In the presence of 35 wt% of graphene oxide as an eco-benign heterogeneous catalyst, β-naphthol, aromatic aldehydes and urea undergo cyclocondensation within 20 to 30 minutes, offering corresponding products in good to excellent yields. Authors also synthesized 1-amidoalkyl-2-naphthols in a one-pot using graphene oxide as a recyclable catalyst. High yields, reduced reaction time, easy work-up and handling, reusability of the catalyst are the advantages of the stated protocol.

### 2.27. Tetramethyl ammonium hydroxide (TMAH) catalyzed protocol

**Scheme 27:**
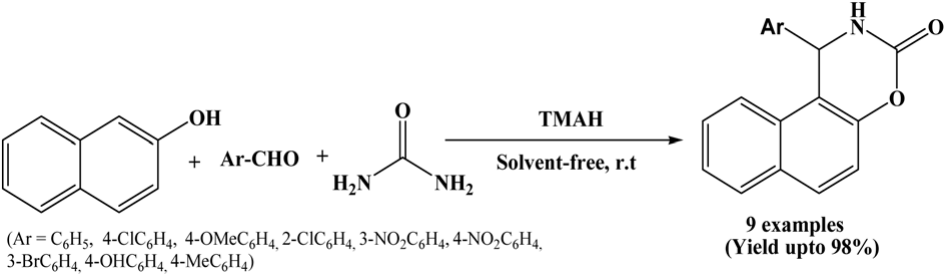
TMAH catalyzed synthesis of naphthoxazinones.

In 2016, Montazeri and Nezhad reported a facile protocol to synthesize 1,2-dihydro-1-arylnaphtho[1,2-e][1,3]oxazine-3-one derivatives through the reaction between β-naphthol, aromatic aldehydes and urea using 35 mol% of tetramethyl ammonium hydroxide (TMAH) as an efficient catalyst. Reactions were completed in 2 h at room temperature under solvent-free conditions (Scheme 27).^44^ The authors investigated the effect of various protic and aprotic solvents in the reaction between 2-naphthol, benzaldehyde and urea in presence of TMAH, and observed decrease in product yield in all cases. Notably, the reaction afforded excellent yield (98%) under solvent-free conditions. The stated protocol offers several advantages such as solvent-free conditions, room-temperature reactions, easy work-up, low cost, use of a readily available catalyst and excellent yields of the desired products.

### 2.28. Catalyzed by iron(iii) phosphate, FePO_4_

**Scheme 28:**
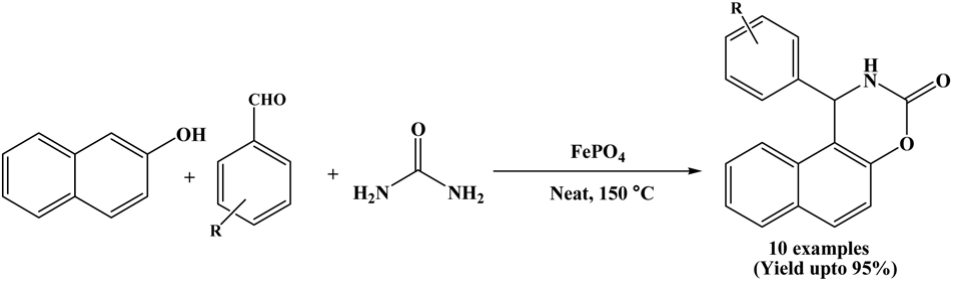
Synthesis of naphthoxazinone derivatives on FePO_4_.

In 2017, Rahmani and K. Behbahani reported the synthesis of 1,2-dihydro-1-arylnaphtho[1,2-e][1,3]oxazine-3-one derivatives applying FePO_4_ as a cost-effective and easily available reagent. The reported cyclocondensation reaction between β-naphthol, aromatic aldehydes and urea with FePO_4_ (20 mol%) afforded the corresponding products in good yields under solvent-free conditions at 150 °C (Scheme 28).^45^ The catalyst could be recovered and reused for three times. Benzaldehyde and a range of aromatic aldehydes bearing both electron-withdrawing and electron-donating substituents were successfully employed, affording the corresponding naphthoxazinones in good yields.

### 2.29. Catalyzed by snail shell

**Scheme 29:**
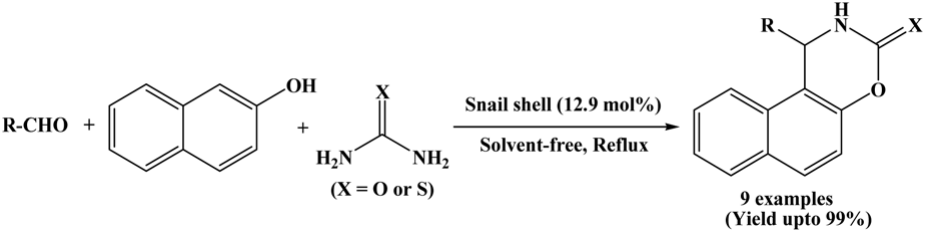
Snail shell mediated synthesis of naphthoxazinone derivatives.

In 2017, Benzekri *et al.* designed a novel method for the synthesis of naphthoxazine-3-one/thione derivatives using snail shell as a green, non-toxic and natural catalyst. One-pot three-component condensation of 2-naphthol, aromatic aldehydes and urea/thiourea was accomplished under solvent-free and reflux conditions using snail shell as a heterogeneous catalyst (Scheme 29).^46^ Reactions were performed in only 5 to 25 minutes. Benzaldehyde and a various aromatic aldehydes containing both electron-withdrawing and electron-donating substituents (R = –H, 4-Cl, 2-Cl, 4-NO_2_, –Me, 2,4-Cl, 4-OMe) were successfully employed, affording the corresponding naphthoxazinones in excellent yields (94 to 99%). The catalyst could be reused for eight runs without any significant loss of catalytic activity.

### 2.30. Microwave-assisted synthesis of napthoxazinones in presence of FeCl_3_

**Scheme 30:**
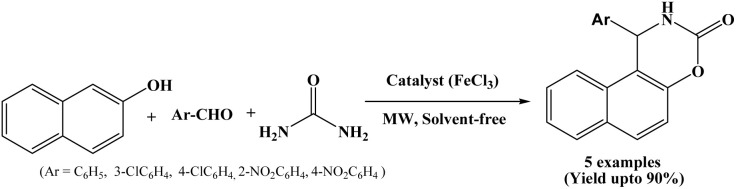
Synthesis of naphthoxazinone derivatives using FeCl_3_.

In 2019, Fardood *et al.* reported an efficient procedure for the synthesis of naphthoxazine-3-ones by one-pot three component condensation of β-naphthol, aryl aldehydes and urea under solvent-free conditions using FeCl_3_ as a catalyst and assisted by microwave irradiation (500 W) (Scheme 30).^47^ Reactions underwent within 15 minutes using 12 mol% anhydrous FeCl_3_ under solvent-free conditions. Various aryl aldehydes with electron-donating and electron-withdrawing groups were evaluated, observed good to excellent yields of the corresponding naphthoxazine-3-one derivatives. The proposed method employed in the synthesis of napthoxazine-3-ones is optimal, cost-effective, and environmentally friendly, in accordance with the principles of green chemistry.

### 2.31. Catalyzed by chitosan

**Scheme 31:**
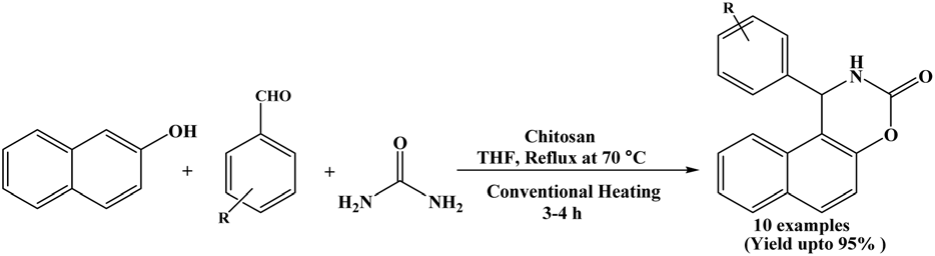
Synthesis of naphthoxazinone derivatives over chitosan.

In 2019, Reddy *et al.* reported a green and efficient one-pot three-component synthesis of 1,2-dihydro-1-arylnaphtho[1,2-e][1,3]oxazine-3-one derivatives using chitosan as a heterogeneous catalyst. In this protocol, 2-naphthol, aromatic aldehydes and urea were reacted under conventional heating conditions at 70 °C in THF solvent in the presence of chitosan to afford the desired naphthoxazine-3-one derivatives in excellent yields (Scheme 31).^9^ Benzaldehyde and a various aromatic aldehydes containing various substituents were successfully employed, affording the corresponding naphthoxazinones in excellent yields (up to 95%). Chitosan, a naturally occurring heteropolymer used as a mild bifunctional heterogeneous and ecofriendly catalyst and could be reused for six successive runs. Owing to its several hydroxyl, amino, and acetamido groups, chitosan has also been investigated as an adsorbent in water purification applications.^48^

### 2.32. Propylphosphonic anhydride (T3P®) catalyzed synthesis

**Scheme 32:**
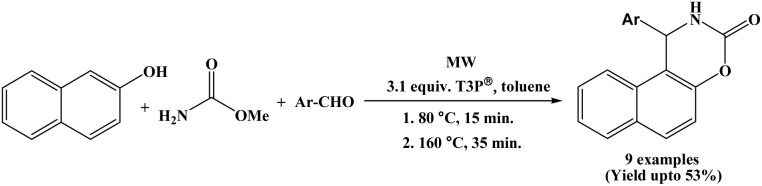
Microwave assisted synthesis of naphthoxazine-3-ones by propylphosphonic anhydride.

In 2020, Varga *et al.* reported an efficient sequential a one-pot two-step protocol for the synthesis of naphthoxazinones from 2-naphthol, methyl carbamate and aromatic aldehydes using a propylphosphonic anhydride in toluene solvent under microwave irradiation (Scheme 32).^49^ In first step, 2-naphthol, methyl carbamate and aromatic aldehydes react at 80 °C, to afford 1-carbamatoalkyl 2-naphthols within 15 minutes. In second step, intramolecular cyclization was carried out at 160 °C for 35 minutes in presence of T3P®, yielding desired products. After establishing the suitable route for the T3P^®^-assisted one-pot synthesis of naphthoxazinones, the scope and limitations of the reaction were investigated with various aromatic aldehydes. Aldehydes bearing substituents such as –H, 3-F, 3-Cl-4-F, 3-OPh, and 3,4-di-F reacted smoothly, affording the corresponding products in moderate yields (41–53%). Aromatic aldehyde with substituent such as 4-OPh produced the desired product in a low yield (23%), which was ascribed to the lower efficiency observed in the first step of the reaction. Notably, when heterocyclic aldehydes were employed, only a trace amount of a side product was isolated in very poor yield, along with unidentified decomposition products, and the desired naphthoxazinone derivatives were detected only by HPLC–MS.

### 2.33. Phenylboronic acid catalyzed synthesis

**Scheme 33:**
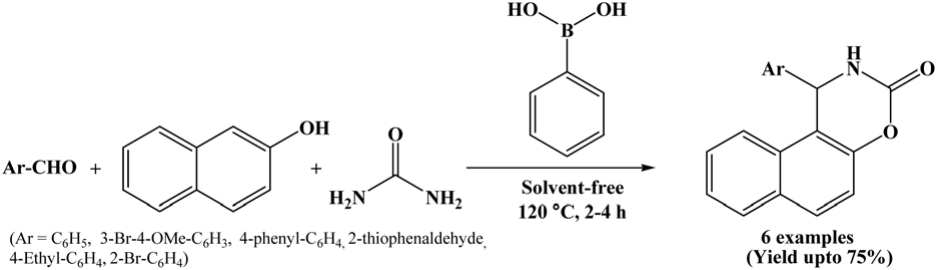
Phenylboronic acid mediated synthesis of naphthoxazinones.

In 2021, Boudebbous *et al.* reports the synthesis and biological evaluation of several naphthoxazine-3-one derivatives. The compounds were synthesized by three-component one-pot condensation reaction of β-naphthol, aromatic aldehydes and urea using phenylboronic acid under solvent-free conditions at 120 °C (Scheme 33).^10^

The synthesized derivatives were then evaluated for their *in vitro* inhibitory activity against cholinesterase (AChE/BChE) and α-glucosidase enzymes, which are important targets for the treatment of neurodegenerative disorders such as Alzheimer's disease and metabolic disorders like diabetes. Some of the synthesized compounds showed promising inhibitory activity. In addition, *in silico* studies were performed to understand the interaction of the synthesized compounds with the active sites of the enzymes, supporting the experimental results and signifying that these naphthoxazinones could serve as potential lead compounds for the new drug development.

### 2.34. Catalyzed by Al/Ag_3_PO_4_

**Scheme 34:**
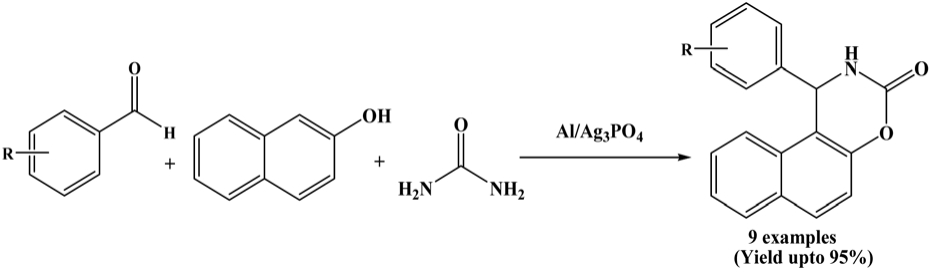
Al/Ag_3_PO_4_ catalyzed synthesis of naphthoxazinones.

El Hallaoui *et al.* in 2021 have utilized a new catalyst namely Al/Ag_3_PO_4_ for the first time as a heterogeneous catalyst for the synthesis of naphthoxazinone derivatives. The new bimetallic phosphate catalyst Al/Ag_3_PO_4_ catalyzed efficiently three-component synthesis of naphthoxazinones from aromatic aldehydes, 2-naphthol and urea under solvent-free and thermal conditions (Scheme 34).^2^ The Al/Ag_3_PO_4_ catalyst was synthesized by a modification of Triple Super Phosphate (TSP) fertilizer with AgSO_4_ followed the impregnation of Al using Al(NO_3_)_3_. The prepared Al/Ag_3_PO_4_ catalyst was characterized by XRD, FT-IR, EDX and SEM spectral techniques. Benzaldehyde and a various aromatic aldehydes containing both electron-withdrawing and electron-donating substituents were successfully employed, affording the corresponding naphthoxazine-3-ones in good to excellent yields (81 to 95%). The corresponding naphthoxazinone derivatives were obtained within few minutes at around 110 °C temperature. The catalyst could be reused up to four runs without significant loss of catalytic activity.

### 2.35. Microwave-assisted synthesis of naphthoxazinones using SiO_2_–ZnCl_2_

**Scheme 35:**
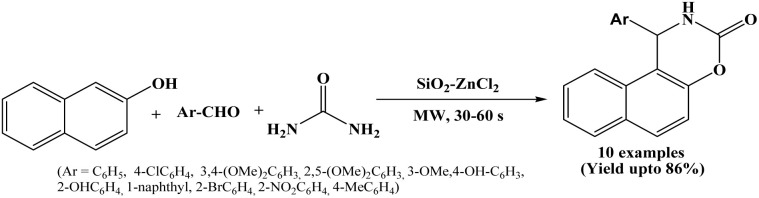
Synthesis of naphthoxazinones over SiO_2_–ZnCl_2_ catalyst.

Microwave-assisted efficient and simple protocol has been reported by Sumi *et al.* in 2025 for the synthesis of naphthoxazine-3-one derivatives using SiO_2_–ZnCl_2_ catalyst. The β-naphthol, aryl aldehydes and urea underwent one-pot multicomponent cyclocondensation reaction to afford naphthoxazine-3-one derivatives under solvent-free conditions and microwave irradiation at 80 °C in the presence SiO_2_–ZnCl_2_ (Scheme 35).^50^ 10 compounds have been synthesized by varying aromatic aldehydes with electron-donating and electron-withdrawing groups within 30 to 60 seconds, with some compounds reported as novel molecules. The catalyst can be recycled and reused up to four successive runs without any notable loss of catalytic activity. The microwave irradiation significantly reduced reaction time while improving product yield with simple work-up procedure.

The synthesized compounds were further evaluated for *in vitro* antioxidant activity, where certain derivatives containing hydroxy and methoxy substituents exhibited notable antioxidant activity, signifying that these naphthoxazinone derivatives may have promising biological applications.

### 2.36. Catalyzed by magnetic nickel-zinc ferrite nanocatalyst (Ni_0.5_Zn_0.5_Fe_2_O_4_, NZF nanocatalyst)

**Scheme 36:**
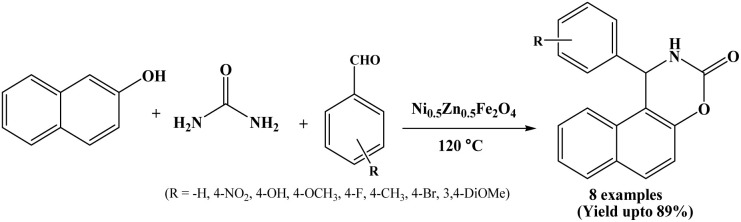
Ni_0.5_Zn_0.5_Fe_2_O_4_ catalyzed synthesis of naphthoxazine-3-ones.

Recently in 2025, Sreekandan *et al.* have performed the synthesis of 1,2-dihydro-1-arylnaphtho[1,2-e] [1,3]oxazine-3-ones in presence of Ni_0.5_Zn_0.5_Fe_2_O_4_ as a magnetic and heterogeneous nanocatalyst under solvent-free conditions at 120 °C (Scheme 36).^51^ The Ni_0.5_Zn_0.5_Fe_2_O_4_ (nickel–zinc ferrite, NZF) nanocatalyst was synthesized by a sol–gel auto combustion method and its structure and morphology were studied using XRD, FT-IR SEM and TEM spectroscopic techniques. The magnetic properties of NZF nanoparticles were also measured. The nickel-zinc ferrite nanocatalyst could be magnetically recovered after the reaction and reused three times without any significant loss of catalytic activity.

## 3. Comparative summary of reported methods for the synthesis of naphthoxazine-3-one derivatives

The synthetic methods for naphthoxazine-3-one derivatives described in Sections 2.1 to 2.36 have been comprehensively summarized in Table 1. This table provides a clear overview of the various strategies, reaction conditions, and purification methods reported across the different synthetic approaches.

**Table 1:** Comprehensive summary of reported methods for the synthesis of naphthoxazine-3-one derivatives

Sr. no.	Catalyst	Reaction conditions	Time	Yield (%)	Purification method	Authors (year)	Ref. n.
1	P-TSA	(I) Solvent-free/160 °C	(I) 1.5 h	(I) 58–64	Recrystallization using EtOAc : hexane (1 : 3)	Dabiri *et al.* (2007)	13
		(II) MW (without any acidic catalyst)	(II) 6 min (for MW)	(II) 69–82 (for MW)			
2	[bmim]Br/p-TSA	[bmim]Br/160 °C	30 min	58–79	Recrystallization using EtOAc : hexane (1 : 3)	Dabiri *et al.* (2007)	15
3	Zn(OTf)_2_	Reflux/CH_3_CN	5–7 h	72–84	Recrystallization using EtOAc : hexane	Hajra *et al.* (2009)	16
4	TMSCl/NaI	CH_3_CN/DMF(4 : 1)/140 °C	3–6 h	62–89	Column chromatography	Sabitha *et al.* (2010)	17
5	Iodine	Solvent-free/80 °C, hot plate	5 min	90–96	Recrystallization using alcohol or column chromatography	Nizam and Päsha (2010)	18
6	Silica-gel	Solvent-free/160 °C	1.5–2 h	70–80	Recrystallization using EtOAc : hexane (1 : 3)	Kottawar *et al.* (2010)	19
7	HClO_4_/SiO_2_	Solvent-free/150 °C	1 h	75–92	Filtration	Ahangar *et al.* (2010)	20
8	Phosphomolybdic acid	DMF/100 °C	2.5–3.5 h	84–92	Recrystallization using 2-propanol	Chaskar *et al.* (2011)	21
9	TMSCl	DMF/135–140 °C	12 h	49–83	Column chromatography	Jiang *et al.* (2010)	22
10	Montmorillonite K10 clay	Solvent-free/160 °C	30–90 min	70–90	Recrystallization using EtOAc : hexane (1 : 3)	Kantevari *et al.* (2010)	23
11	Copper NPs	PEG-400/r.t. K_2_CO_3_, air	45–60 min	74–93	Recrystallization using EtOAc : hexane (1 : 3)	Kumar *et al.* (2011)	5
12	Thiamine hydrochloride	Solvent-free/150 °C	30 min	83–94	Recrystallization using EtOAc : hexane (1 : 3)	Lei *et al.* (2011)	24
13	ZnO NPs	Solvent-free/150 °C	45–120 min	76–94	Filtration	Dharma Rao *et al.* (2012)	28
14	FeCl_3_/nano-SiO_2_	Solvent-free/150 °C	7–20 min	78–95	Recrystallization using MeOH	Safaei-Ghomi *et al.* (2012)	29
15	Wet cyanuric chloride	Solvent-free/150 °C	2–15 min	71–97	Recrystallization using EtOH	Nemati and Beyzai (2013)	30
16	Silica supported Pressler heteropoly acid	C_2_H_5_OH/Reflux	30–130 min	70–92	Recrystallization using CH_2_Cl_2_	Gharib *et al.* (2013)	31
17	Guanidine hydrochloride	Solvent-free/140 °C	60–75 min	85–90	Precipitation using EtOH, followed by filtration	Olyaei *et al.* (2013)	32
18	Pyridinium-based ionic liquid, [PSPy][HSO4]	Solvent-free/150 °C	60 min	76–91	Filtration	Dong *et al.* (2013)	33
19	TiCl_4_	(I) Solvent-free/120 °C	(I) 50–70 min	(I) 60–95	Recrystallization using EtOAc:hexane (1 : 3)	Hunnur *et al.* (2017)	34
		(II) MWI	(II) 5–10 min	(II) 78–96			
20	Silica bonded vanadic acid	Solvent-free/130 °C	45–60 min	81–88	Recrystallization using ethanol/water (10 : 1)	Ali Zolfigol *et al.* (2015)	35
21	Fe_2_O_3_ NPs	PEG-400/r.t. K_2_CO_3_, air	40–60 min	91–95	Recrystallization using EtOAc : hexane (1 : 3)	Hashemzehi-Goonaki and Saffari (2015)	4
22	Silica-bonded S-sulfonic acid	Solvent-free/150 °C	45–90 min	80–94	Column chromatography	Niknam and Abolpour (2015)	36
23	CuFe_2_O_4_ NPs	PEG-400/r.t. K_2_CO_3_, air	25–35 min	89–95	Recrystallization using EtOH	Ghanni and Saffari (2016)	37
24	Perlite-SO_3_H NPs	(I) Solvent-free/110 °C	(I) 40–60 min	(I) 45–65	Recrystallization using aqueous EtOH	Ramazani *et al.* (2016)	38
		(II) MW	(II) 2–10 min	(II) 79–92			
25	Nano-TiO_2_	Solvent-free/130 °C	30 min	70–90	Recrystallization using 2-propanol	Khalil Pourshamsian (2018)	42
26	Graphine oxide	Solvent-free/120 °C	20–30 min	85–89	Filtration	Gupta *et al.* (2016)	43
27	Tetramethyl ammonium hydroxide (TMAH)	Solvent-free/r.t.	2 h	89–98	Recrystallization using ethanol	Montazeri and Mohammad Nezhad (2016)	44
28	Iron(iii) phosphate, FePO_4_	Solvent-free/150 °C	90–240 min	75–95	Recrystallization using EtOH	Rahmani and K. Behbahani (2017)	45
29	Snail shell	Solvent-free/Reflux	5–25 min	94–99	Recrystallization using EtOH	Benzekri *et al.* (2017)	46
30	FeCl_3_	Solvent-free/MW	15 min	84–90	Recrystallization using EtOH	Taghavi Fardood *et al.* (2019)	47
31	Chitosan	THF/Reflux at 70 °C	3–4 h	90–95	Column chromatography	Narasimha Reddy *et al.* (2019)	9
32	Propylphosphonic anhydride	Toluene/MW	35 min	41–53	Column chromatography	Varga *et al.* (2020)	49
33	Phenylboronic acid	Solvent-free/120 °C	2–4 h	60–75	Column chromatography	Boudebbous *et al.* (2021)	10
34	Al/Ag_3_PO_4_	Solvent-free/110 °C	10–20 min	81–95	Recrystallization using EtOH	Hallaoui *et al.*(2021)	2
35	SiO_2_–ZnCl_2_	Solvent-free/microwave irradiation at 80 °C	30–60 seconds	76–86	Column chromatography	Sumi *et al.* (2025)	50
36	Nickel–zinc ferrite nanocatalyst	Solvent-free/120 °C	120 min	85–89	Column chromatography	Sreekandan *et al.* (2025)	51

## 4. Challenges and future perspectives in the synthesis of naphthoxazine-3-ones

Although the synthesis of naphthoxazinones has advanced considerably over recent years, several challenges and research gaps are there to limit the broader development and applications of naphthoxazine-3-one derivatives. In most of the reported protocols by various groups of researchers, it is observed that naphthoxazine-3-one derivatives were synthesized under solvent-free and thermal conditions at temperature up to 160 °C. Limited functional group tolerance observed in many reported protocols. Expanding substrate compatibility to include a broader range of the functional groups is essential, particularly for applications in drug discovery where molecular diversity plays a significant role. Another significant gap is the lack of stereoselective and regioselective strategies. Control over selectivity is crucial for generating structurally defined compounds with potential biological relevance.

From a medicinal chemistry perspective, there is also a clear disconnect between synthetic development and biological evaluation. While numerous naphthoxazinone derivatives have been synthesized, systematic studies exploring their pharmacological properties, structure–activity relationships, and mechanisms of action remain rare. This gap hinders the identification of lead compounds and delays their translation into potential therapeutic agents.

The present review aims to address these research gaps by providing a comprehensive and critical overview of the existing synthetic methodologies for the synthesis of naphthoxazine-3-ones. By systematically comparing reaction conditions, catalysts, yields, and substrate scope, this review highlights the strengths and limitations of current approaches. Importantly, it brings attention to areas such as more green synthetic strategies, more novel catalytic systems, and the need for stereo control synthesis.

Moreover, this review attempts to bridge the gap between synthetic chemistry and medicinal applications by summarizing the limited but promising biological studies associated with naphthoxazinone frameworks and related heterocycles. By identifying trends in the literature, it offers clear directions for future research, including the development of more sustainable and scalable methods, expansion of chemical diversity, and integration of biological screening early in the design process.

There are several reports on the synthesis of naphthoxazine-3-ones by conventional catalytic approaches, including thermal and microwave-assisted methods. However, to the best of our knowledge, there are no reports describing the photocatalytic synthesis of naphthoxazine-3-ones. This highlights a potential research gap and suggests that the development of photocatalytic strategies could represent a promising future direction in the synthesis of naphthoxazine-3-one derivatives.

Future research in the synthesis of naphthoxazinones is expected to emphasize the development of sustainable and scalable synthetic methodologies, along with enantioselective approaches that improve efficiency and atom economy. Looking ahead, researchers should place greater emphasis on molecular docking to identify the most effective naphthoxazinone scaffolds. Designing and synthesizing compounds based on these insights, followed by thorough biological evaluation, could improve their chances of success in pharmaceutical applications. In addition, the exploration of other strategies such as photochemical, flow chemistry, and electrochemical methods represents a promising and relatively unexplored methods for the synthesis of naphthoxazine-3-one derivatives.

In summary, this review contributes meaningfully to the field of synthesis of naphthoxazine-3-one derivatives by clarifying existing limitations, organizing current knowledge, and outlining practical directions for future research.

## 5. Conclusions

In this review, we have attempted to highlight the numerous catalytic systems used in the synthesis of naphthoxazine-3-one derivatives by one-pot three-component condensation of β-naphthol, aromatic aldehydes and urea. In most of the reported protocols by various groups of researchers, it is observed that naphthoxazin-3-one derivatives were synthesized under solvent-free and thermal conditions at temperature up to 160 °C. In few reports, synthesis of naphthoxazine-3-ones have been caried out at room temperature also. In few reports, naphthoxazinone derivatives were synthesized using solvents like PEG-400, DMF, THF, toluene, CH_3_CN and [bmim]Br. In some protocols, corresponding compounds were synthesized by microwave assisted methods, with substantial decrease in reaction time and increase in yields. Nanostructured catalytic systems were also employed efficiently in many protocols for the synthesis of naphthoxazinone derivatives. We have also highlighted few methods in which naphthoxazinone derivatives were synthesized from β-naphthol, aromatic aldehydes and methyl carbamate. Most of the reported protocols have significant advantages like simple procedure, easy work-up, shorter reaction time, solvent-free conditions, purification of compounds by simple recrystallisation method, use of non-toxic and inexpensive catalyst, good to excellent yields of the desired products, recyclability and reusability of the catalysts, making green and environmentally benign protocols. This review will be definitely valuable to researchers working on the synthesis of naphthoxazinone derivatives and related compounds, aiding to the development of more efficient and environmentally friendly protocols.

## Conflicts of interest

The authors declare no conflicts of interest.

## Data Availability

No new data were generated in this review. All data discussed are available in the cited references.
